# Beta-Blockers for the Secondary Prevention of Myocardial Infarction in People with Dementia: A Systematic Review

**DOI:** 10.3233/JAD-190503

**Published:** 2019-10-15

**Authors:** David Lanham, Sana Ali, Daniel Davis, Mark James Rawle

**Affiliations:** a MRC Unit for Lifelong Health and Ageing at UCL, London, UK; b Barts and The London School of Medicine and Dentistry, London, UK

**Keywords:** Beta-blockers, dementia, myocardial infarction, secondary prevention, systematic review

## Abstract

**Background:**

Cardiovascular disease remains the most common cause of death in industrialized countries. The use of beta-blockers is well established as a secondary prevention of myocardial infarction. However, little is known about the benefits of beta-blockers for people living with dementia.

**Objective:**

To evaluate the use of beta-blockers in people with dementia who have had a myocardial infarction, in order to identify associations between medication use, mortality, re-infarction and functional decline.

**Methods:**

We searched for all studies (randomized trials, observational cohorts) reporting beta-blocker use in populations with both dementia and previous myocardial infarction. Relevant keywords were used in Medline, Embase, and Web of Science up to October 2018. Titles and abstracts were independently screened by two reviewers. Quality of eligible studies was assessed using the Newcastle-Ottawa Scale. PRISMA recommendations were followed throughout.

**Results:**

Two observational studies were included, representing 10,992 individuals in a community setting and 129,092 individuals from a hospital record-linkage study. One showed use of beta-blockers reduced all-cause mortality (HR 0.74 (95% CI 0.64– 0.86) alongside evidence for an increased rate of functional decline in individuals aged≥65 with moderate to severe cognitive impairment (OR 1.34 (95% CI 1.11– 1.61)). The second study did not find an association between beta-blocker use and mortality in the population living with dementia.

**Conclusion:**

There is insufficient evidence to support use of beta-blockers to persons living with dementia. A single study provides limited evidence that beta-blockers improve survival rates but with associated detrimental effects on functional status in nursing home residents with cognitive impairment. Decisions to continue beta-blockers in persons living with dementia should be made on an individual basis.

## INTRODUCTION

Cardiovascular disease remains the most common cause of death in industrialized countries [1]. Advances in both primary and secondary prevention of myocardial infarction (MI) have had a significant positive impact on reducing morbidity and mortality from cardiovascular disease. Beta-blockers are well-established for pharmacological secondary prevention [2], and have been found to reduce mortality when used in post MI in the general population [3, 4].

The evidence for the use of beta-blockers is predominantly drawn from clinical trials excluding persons living with dementia [5]. This is despite the fact that cardiovascular disease and dementia commonly co-exist. A 2017 estimate found 850,000 people in the UK living with dementia [6] with concomitant diagnoses of ischemic heart disease found in almost a quarter of people with dementia [7]. In prior analyses of approximately 12,000 individuals in the UK’s General Practice Research Database, prescriptions of beta-blockers were found in almost 20% of persons with Alzheimer’s disease or vascular dementia, with highest levels found in the latter group [8].

In a population living with dementia, any potential benefit from pharmacotherapy must be offset by potential risks. Persons living with dementia have higher rates of hospitalization, shorter life expectancy [9], and are often on multiple medications [10], where concordance can be difficult, polypharmacy is costly and side-effects and drug interactions unknown. Such adverse outcomes may be undetected in the context of a clinical trial [11]. Furthermore, life expectancy of those with dementia may be too short to yield benefit from pharmacological secondary prevention. Polypharmacy is also associated with reduced physical and cognitive function [12] and the complexity of medication regimens has been linked to functional impairment due to medication administration errors [10], themselves more common in those with cognitive impairment [9]. Given these issues, existing randomized controlled trials are limited in their generalizability for persons living with dementia.

In order to address the question of whether beta-blockers might continue to be beneficial in persons living with dementia, we performed a systematic review to assess the quantity and quality of evidence supporting beta-blocker therapy as pharmacological secondary prevention for persons living with dementia.

## METHODS

### Objectives

We set out to answer the following question: is there any evidence to support the role of beta-blockers in the secondary prevention of MI in individuals with established dementia? A secondary objective involved identifying the risks and benefits of beta-blocker use in this population. We searched for all types of study design, that is, both randomized controlled trials and observational studies.

### Inclusion criteria

#### Population

Peer-reviewed studies published since 1965 (when beta-blockers were first used in the secondary prevention of MI [13]) that included persons with previous MI (including STEMI and NSTEMI) and also reporting of any associations in individuals with a diagnosis of dementia/cognitive impairment.

#### Intervention

Both interventional and observational studies investigating beta-blocker use.

#### Outcomes

Studies reporting mortality/survival, re-infarction rates, functional decline, and/or serious adverse events related to medication use.

#### Analysis

Any quantification of associations between these outcomes and beta-blocker use.

### Exclusion criteria

We excluded any non-English language articles

### Search strategy

We identified publications by first developing search terms through an exploratory Medline search, recording relevant keywords found in the title, abstract, and Medical Subject Heading (MeSH) terms. This informed a comprehensive search using the relevant keywords and synonyms on Medline, Embase, and ISI Web of Science databases (Supplementary Material). Keywords used were ‘beta’, ‘ischaemic heart disease’, and ‘dementia’, along with synonyms and variants. A comprehensive list of prescribed beta-blockers was obtained from the British National Formulary and were included in the search. No filters were used, searching from 1965 up to October 20, 2018. We did not identify any existing protocols through our search or registered on PROSPERO. No de-duplication was performed, and all abstracts were independently reviewed by two reviewers (DL, SA) with disagreements resolved by consensus. Full texts were considered further and data extracted from included studies using a pro forma.

### Data abstraction and validity assessment

For each of the studies meeting the inclusion criteria, we extracted data of interest including: age, sex, total number of participants, number who had cognitive impairment, and number of study participants on beta-blockers. We used the Newcastle-Ottawa assessment scale to assess risk of bias and evaluate study quality (Supplementary Table 1) [14]. The principal summary measures of interest were recorded as odds ratios, risk ratios, and hazard ratios. We did not identify studies of sufficient similarity to undertake a meta-analysis.

## RESULTS

**Fig. 1:**
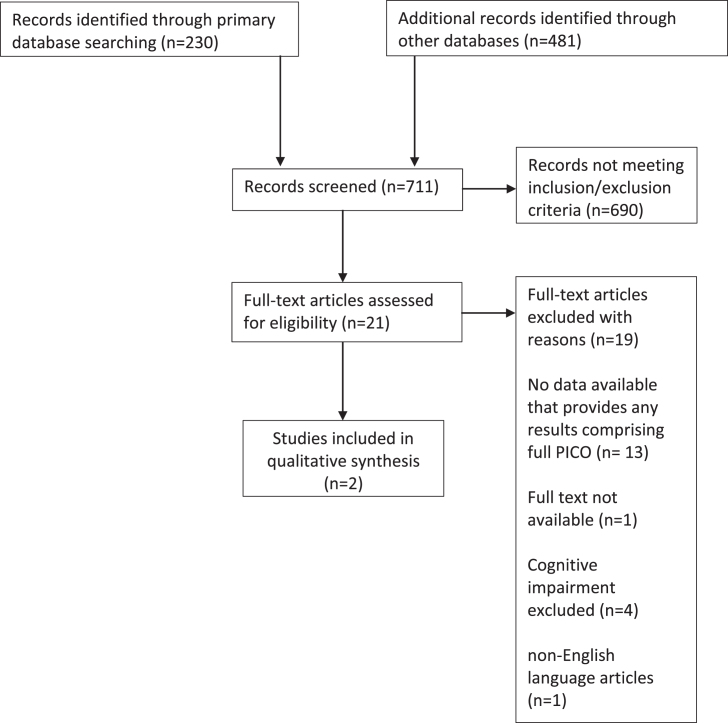
Study flow diagram.

The initial search provided 230 abstracts from Pubmed, 363 from Embase, and 118 from ISI Web of Science (Fig. 1).

We identified twenty-one articles that met inclusion criteria and merited full-text review (Table 1). After applying exclusion criteria, only two directly quantified beta-blocker use in relation to our outcomes of interest in individuals with dementia [15, 16]. The first study investigated dementia status (as documented in hospital notes) in relation to outcomes in Medicare beneficiaries admitted to hospital with an acute MI between 1994-1995 (Medicare study). The second study included US nursing home residents post-acute MI examining associations between beta-blockers and functional, mortality, and rehospitalization outcomes between 2007– 2010 (Nursing Home study). This study grouped patients by their score on the Cognitive Performance Scale as having either normal cognition, mild-moderate dementia, or moderately severe-very severe dementia. Both were secondary analyses of electronic health records. Mortality was the primary outcome measure in both studies. The characteristics of both studies are detailed in Table 2. The two studies received a Newcastle-Ottawa scoring of 7 and 8 with a maximum obtainable score of 9 indicating ‘fair’ to ‘good’ study design and methodology.

**Table 1:** Papers eligible for full screening

Study title	Type of study	Average age	Gender split M/F	Total no participants	No participants cognitive impairment	No on beta-blockers	Was association made	Comments
Differences in management and outcomes for men and women with ST-elevation myocardial infarction [22].	Prospective Cohort Study	63	2183/715	2898	67	2370	NO	Review of STEMI management between gender, does not review relationship between beta-blockers and patients with dementia
Secondary Prevention Medication Use After MYOCARDIAL INFARCTION in U.S. Nursing Home Residents [23].	Retrospective cohort study	84	3165/8027	11192	9348	6369	NO	does not directly investigate beta blocker use and unclear if all secondary prevention medication was commenced.
The prescription of antiplatelet medication in a very elderly population: An observational study in 15 141 ambulatory subjects [24].	Retrospective observational	86	5860/9281	15141	1188	5955	NO	Had data on patients with cognitive impairment and who took beta-blockers but no way of inferring between the two as direct association not investigated
Association of *β*-BLOCKERs with Functional Outcomes, Death, and Rehospitalization in Older Nursing Home Residents After Acute Myocardial Infarction [16].	Propensity matched cohort	84	3204/7788	10992	3916	5496	YES	Associated with increased functional decline, but lower mortality rates
Blood Pressure Lowering Medication, Visit-to-Visit Blood Pressure Variability, and COGNITIVE Function in Old Age [25].	Data from PROSPER RCT	///	///	///	///	///	EXCLUDE	patients with cognitive impairment were excluded at start
Ischemic heart disease, prescription of optimal medical therapy and geriatric syndromes in community-dwelling older men: A population-based study [26].	Prospective Cohort Study	77	1694/0	1694	214 of which 59 had IHD	375 of which 191 had IHD	NO	looked at participants with IHD who had cognitive impairment and with IHD on beta-blocker, but no comparisons drawn
The design and rationale of a multicenter clinical trial comparing two strategies for control of systolic blood pressure: The Systolic Blood Pressure Intervention Trial (SPRINT) [27].	Multicenter RCT	///	///	///	///	///	EXCLUDE	patients with cognitive impairment were excluded at start
Effect of DEMENTIA on the use of drugs for secondary prevention of ischemic heart disease [28].	retrospective cohort analysis	76.6	567/520	1087	265	229 – (8 with dementia)	NO	does not look at effect of beta-blocker use on outcome
Prevalence and correlates of cardiovascular medication use among nursing home residents with ischemic heart disease: results from the SHELTER study. [29]	retrospective cohort analysis	∼85	286/764	1050	693	353	NO	notes participants who have dementia, no specific numbers on comparing beta-blocker and no beta-blocker with those who do have dementia and no mention of outcome.
Mid-term mortality of very elderly patients with acute MYOCARDIAL INFARCTION with or without coronary intervention [30].	Observational study	∼85	41/36	77	10	22	NO	comparing PCI to no PCI with different outcomes
COGNITIVE function and antihypertensive treatment in the elderly: a 6-year follow-up study [31].	Follow up study	77	∼	518	?	61	EXCLUDE	Although association drawn between MMSE and beta-blocker use, not a baseline cognitive impaired cohort and not a previous MI cohort
Effects of cardiovascular medications on rate of functional decline in Alzheimer disease [32].	prospective Cohort Study	∼86	N/A	216	216	33	NO	did not associate whether participants had previous MI and use of beta-blocker in the outcome
A review of the management of heart failure in long-term care residents [33].	cross sectional study	83.2	98/207	302	Not known	Not Known	NO	UNABLE TO OBTAIN FULL PAPER but abstract making no suggestion of association being made as only 30% of patients had either IHD OR dementia
Association between functional status and use and effectiveness of beta-blocker prophylaxis in elderly survivors of acute myocardial infarction [34].	cross sectional/retrospective study	75	24645/20695	45730	2143	22683	NO	only 25% IHD, 8000 prescribed beta-blocker prior to admission, looks at prescription with outcome, but not associated with dementia
The effect of dementia on outcomes and process of care for Medicare beneficiaries admitted with acute myocardial infarction [15].	Retrospective chart review	75	68637/60455	129092	5851	39556	YES	mortality higher in dementia patients but proportion on beta-blockers same across groups
[Use of diagnostic and therapeutic resources in patients hospitalized for heart failure: influence of admission ward type (INCARGAL Study)] [35].	Cross sectional study	///	///	///	///	///	EXCLUDE	article in Spanish
Multifactorial cardiovascular disease prevention in patients aged 75 years and older: A randomized controlled trial: Drugs and Evidence Based Medicine in the Elderly (DEBATE) Study [36].	RCT	///	///	///	///	///	EXCLUDE	No clear data on patients with cognitive impairment
Occurrence and progression of DEMENTIA in a community population aged 75 years and older: relationship of antihypertensive medication use [37].	Cohort study	82.5	514/1296	1810	224	Not known	NO	No clear data on which patients had established MI
Beta-blocker Use in U.S. Nursing Home Residents After Myocardial Infarction: A National Study [38].	Retrospective cohort study	83	4580/11140	15720	12797	8953	NO	Does not compare mortality between dementia and non-dementia patients with and without beta blockade
Outcomes of Acute Myocardial Infarction in Nonagenarians [39].	Retrospective chart review	93	60/117	177	41	158	NO	Does not compare mortality between dementia and non-dementia patients with and without beta blockade
The impact of DEMENTIA on the outcomes of treatments for acute coronary syndrome [40].	Retrospective cohort study	66	139993/72117	212110	Not known	Not known	NO	Does not identify effect of beta-blocker alone on outcome in dementia versus non dementia patients

IHD, ischemic heart disease; MI, myocardial infarction; MMSE, Mini-Mental State Examination; PCI, percutaneous coronary intervention; RCT, randomized controlled trial.

**Table 2:** Eligible studies included in systematic review

Citation	Study design	Sample	Setting	Data collection	Outcome measures	Co-variates	Summary findings	Quality
Sloan FA, Trogdon JG, Curtis LH, Schulman KA. The effect of dementia on outcomes and process of care for Medicare beneficiaries admitted with acute myocardial infarction [15].	Retrospective cohort study	Any Medicare users with or without dementia admitted for an acute myocardial infarction between 1994 and 1995 (*n* = 129,092)	USA	Medical record review, noting use of beta-blockers and other secondary preventative measures	30 day and 1-year mortality	age, sex, admission source, co-morbidities, and severity of cardiac illness.	Crude differences in percentage taking beta-blockers with respect to mortality (31.1% no dementia versus 21% dementia *p*≤0.01 However overall differences in mortality accounted for by differences in ACE and interventions accounted for in multivariate analysis and regression analysis showed little to no difference of beta-blocker on mortality.	FAIR rating 7 Stars -Newcastle-Ottawa assessment
Steinman MA, Zullo AR, Lee Y, Daiello LA, Boscardin WJ, Dore DD, et al. Association of B-blockers with functional outcomes, death, and rehospitalization in older nursing home residents after acute myocardial infarction [16].	Retrospective Cohort Study	Nursing home residents over 65 who had been admitted to hospital with an acute myocardial infarction in the USA between 2007 and 2010 (*n* = 10,992)	Nursing Home, USA	National data from Minimum data set 2.0 and Medicare Parts A and D which includes assessments of nearly all nursing home residents in USA	90-day mortality, functional decline and rehospitalization.	Propensity Scoring (key co-variates: Baseline functional status, cognitive function, age, presence or absence of an intensive care unit or cardiac care unit stay during the AMI hospitalization)	Decreased risk of death at 90 days HR 0.74 (95% CI 0.64– 0.86) among individuals on beta-blockers. Functional decline in patients with moderate to severe cognitive impairment and who were on a beta-blocker OR 1.34 (95% CI 1.11– 1.61).	GOOD rating 8 Stars– Newcastle-Ottawa assessment

### Mortality

The Medicare study described an increase in mortality of individuals with dementia at 30 days (RR = 1.16; 95% CI, 1.09– 1.22) and one year follow up (RR = 1.18; 95% CI = 1.13– 1.23) compared with the non-dementia group. Participants with dementia were less likely to be on beta-blockers when compared to participants without dementia; though no direct effect size was calculated for the effect of beta-blocker use on mortality in either of these subgroups. However, concurrent lower ACE inhibitor use and fewer cardiac interventions (thrombolysis, catheterization, coronary angioplasty) in those with dementia raise the possibility these factors, either in isolation or in combination with beta-blocker under-use account for the observed higher mortality, rather than beta-blocker use alone. The Nursing Home study showed the use of beta-blockers to reduce mortality (HR = 0.74; 95% CI, 0.67– 0.83) in the cohort as a whole, but also in the subgroup with moderate/severe cognitive impairment (HR = 0.74; 95% CI, 0.64– 0.86). For the study population as a whole, the reported number needed to treat was 26 (95% CI, 19– 39).

### Reinfarction rates and cardiovascular morbidity

No evidence was available from either study on the association of beta-blockers on the rate of subsequent myocardial infarction or consequent cardiovascular morbidity in persons living with dementia.

### Negative sequelae: Hospital readmission, adverse drug reactions, and functional decline

The Nursing Home study reported that beta-blockers were not associated with increase in readmission from a nursing home, irrespective of dementia status (OR 1.06; 95% CI, 0.98– 1.14). The same study also showed more functional decline in individuals with moderate to severe cognitive impairment who were also on a beta-blocker (OR 1.34; 95% CI, 1.11– 1.61)), where functional decline was defined as a loss of 3 points on the Morris Scale of Independence in Activities of Daily Living (ADLs) [17] in the first three months post-hospital discharge. This relates to a number-needed-to-harm as 36 (95% CI, 24– 76). This functional decline was not seen in individuals with intact cognition or mild dementia (OR 1.03; 95% CI, 0.89– 1.20). No negative sequelae were described in the Medicare study, and neither study specifically reported rates of adverse drug reaction.

## DISCUSSION

In two studies of 10,992 individuals in a nursing home setting and 129,092 Medicare recipients, we found weak evidence of beta-blockers being associated with lower mortality in individuals with dementia. However, associations between beta-blocker use and worse functional outcomes in persons living with moderately-severe or worse dementia were also evident. Taken together, our findings suggest that despite the widespread practice of beta-blocker prescription for secondary prevention in persons living with dementia, there is minimal evidence to support any benefit, and potential evidence of harm.

The findings from our systematic review should be interpreted with caution given the paucity of evidence identified. Included studies were observational cohorts, rather than randomized trials. Neither reported follow-up data beyond 12 months, when beta-blockers may have mortality benefit, at least in non-cognitively impaired populations [18]. In addition, neither paper reported re-infarction rates, reduction of which is a key benefit of beta-blocker use in younger populations [19]. Another limitation is that the dementia diagnoses in both studies relied on administrative data, which is likely to under-ascertain cases. Any resultant misclassification bias might then obscure true effects of beta-blockers in this group.

Use of beta-blockers as secondary prevention of MI in the general population dates back to the 1960 s [5]. In 1999, the best contemporary meta-analysis on beta-blockers and all-cause mortality noted an overall annual survival benefit, but no evidence after 1982 that beta-blockers directly led to differences in mortality [5]. With effective angioplasty and catheter revascularization now routine within 24 hours of MI [20, 21], the attributable fraction from beta-blockers on secondary outcomes may have decreased over time. How such trends may differentially affect older people with or without dementia has yet to be established. Indeed, the finding of associations between beta-blockers and high rates of functional decline in the Nursing Home paper may be an example of this. Beta-blocker use may reduce mortality in this group and prolong the lives of individuals who are likely to experience severe functional decline due to their dementia. Equally, this group may be more prone to side effects from beta-blocker use and experience more frequent falls or hypotensive episodes that in turn lead to physical and functional deconditioning.

We highlight the paucity of evidence on beta-blocker use in persons living with dementia. Further studies might include randomized trials, all of which should incorporate a range of outcome measures beyond mortality (secondary cardiac events, functional status, frailty, quality of life). Moreover, there is need to account for people living with dementia at all severities and settings as those with mild dementia living independently may have little in common with more advanced disease in institutional care. At best, beta-blocker use should be considered on a patient by patient basis with explanation to the patient and any carers of a possible mortality benefit, balanced against the possible harm to the functional status. This could result in a reduced quality of extended life. This would be explained with a view to patient and family empowerment in decision making around stopping versus continuing therapy.

## Supplementary Material

Supplementary Material
