# Supplementary material for: Beta-Blockers for the Secondary Prevention of Myocardial Infarction in People with Dementia: A Systematic Review
Source: J Alzheimers Dis. 2019 Oct 15;71(4):1105–14. doi: 10.3233/JAD-190503 (PMC6839460; doi:10.3233/JAD-190503)
Supplement: Supplementary Material [file jad-71-jad190503-s001.docx]

**Supplementary Material**

**Beta-Blockers for the Secondary Prevention of Myocardial Infarction in People with Dementia: A Systematic Review**

1. IHD OR Ischaemic Heart Disease OR MI OR CAD OR Coronary Artery Disease OR cardiovascular disease OR heart attack OR myocardial infarction OR cardiac ischaemia OR coronary ischaemia OR coronary infarction OR STEMI OR NSTEMI OR ACS OR unstable angina OR Acute Coronary Syndrome

2. beta-blocker* OR b-blocker* OR beta-blocker* OR atenolol OR metoprolol OR pindolol OR timolol OR oxprenolol OR nebivolol OR nadolol OR labetalol OR celiprolol OR carvedilol OR bisoprolol OR acebutolol OR propranolol OR alprenolol OR adrenergic beta-antagonist* OR xamoterol OR sotalol OR practolol OR bisoprolol

3. Dementia OR Cognitive impairment OR cogniti* disorders OR delirium OR forgetfulness OR memory impairment OR alzheimers OR lewy body

**Supplementary Table 1.** Newcastle-Ottawa assessment of observational study quality

| **Study** | **Representativeness of exposed cohort** | **Selection of the non-exposed cohort from the same source as the exposed cohort** | **Ascertainment of Exposure** | **Demonstration that outcome of interest was not present at the start of study** | **Comparability of cohorts** | **Assessment of outcome** | **Follow-up long enough for outcome to occur** | **Adequacy of Follow-up** | **Quality Score** |
| --- | --- | --- | --- | --- | --- | --- | --- | --- | --- |
| Sloan et al. (Medicare Study) [15] | Participants were truly representative of the Medicare patients with acute MI in USA between 1994-1995 due to large sample size >200 thousand obtained from 46 states. Taken from 2 data abstraction centers with high degree of consistency in abstraction. *(star)* | Same source  *(star)* | Secure record  *(star)* | All outcomes defined post study entry *(star)* | Blocks of explanatory variables were included for sociodemographic characteristics (including age and sex), admission source, co-morbidities and severity of cardiac illness. - Simple regression but not propensity matching performed. | From records on same two data sources, record linkage performed *(star)* | One year follow up with large sample size *(star)* | All participants results were noted but a significant number of records had missing information. | Selection  = 4*(star)*  Comparability =1 *(star)*  Outcome  =2 *(star)*  Overall rating = **FAIR** |
| Steinman et al.  (Nursing Home Study) [16] | Participants were truly representative of NH residents in the USA as they obtained data from OSCAR which assesses nearly all nursing homes in the USA and is updated at a minimum every three months. *(star)* | Same source  *(star)* | Secure record used as national database  *(star)* | Comparison of start and end point for each patient *(star)* | Propensity scoring was used to assess for 93 variables to predict beta-blocker use, and this yielded excellent covariate balance. *(star)* | From record on same database *(star)* | 3 month and large enough sample size to produce statistically significant results | All participants were followed up but individuals who died or were re-hospitalized at 14 days were excluded *(star)* | Selection  = 4 *(star)*  Comparability = 2 (*star)*  Outcome  = 2*(star)*  Overall rating  = **GOOD** |
